# Consciousness: a neural capacity for objectivity, especially pronounced in humans

**DOI:** 10.3389/fpsyg.2014.00223

**Published:** 2014-03-17

**Authors:** Anton J. M. Dijker

**Affiliations:** Faculty of Health, Medicine and Life Sciences, Maastricht UniversityMaastricht, Netherlands

**Keywords:** consciousness, human evolution, vulnerability, intelligence, conscience, esthetic experience, tool making, mindfulness

## Abstract

Consciousness tends to be viewed either as subjective experience of sensations and feelings, or as perception and internal representation of objects. This paper argues that neither view sufficiently acknowledges that consciousness may refer to the brain’s most adaptive property: its capacity to produce states of objectivity. It is proposed that this capacity relies on multiple sensorimotor networks for internally representing objects and their properties in terms of expectancies, as well as on motivational and motor mechanisms involved in exploration, play, and care for vulnerable living and non-living objects. States of objectivity are associated with a very special phenomenal aspect; the experience that subjective aspects are *absent* and one is “just looking” at the world as it really is and can be. However, these states are normally closely preceded and followed by (and tend to be combined or fused with) sensations and feelings which are caused by activation of sensory and motivational mechanisms. A capacity for objectivity may have evolved in different species and can be conceived as a common basis for other elusive psychological properties such as intelligence, conscience, and esthetic experience; all three linked to crucial behaviors in human evolution such as tool making, cooperation, and art. The brain’s pervasive tendency to objectify may be responsible for wrongly equating consciousness with feelings and wrongly opposing it to well-learned or habitual (“unconscious”) patterns of perception and behavior.

## INTRODUCTION

Consciousness not only tends to be seen as one of the most important and adaptive, but also most elusive properties that brains can ever acquire. This paper argues that its elusiveness in large part is due to a failure to clearly distinguish and characterize the two main aspects that are usually associated with consciousness; on the one hand, feelings, sensations, and experiences, on the other, perception and internal representation of objects and their properties. It is proposed that a clearer view on its adaptive nature is possible by treating consciousness as a capacity for objectivity.

First consider the association between consciousness and perception or internal representation of objects. Many theorists equate consciousness and perception, saying that consciousness has intentionality or is about objects (we are conscious *of* objects), and that perception differs from mere sensation in that its content consists of objects of which we are aware (e.g., James, 1892/1985; Husserl, 1907/1991; Hochberg, 1988; Coren, 2003). Importantly, with a few exceptions, most theorists assume that perception of objects and their invariant properties requires the formation and use of internal representations or expectancies (Hochberg, 1988). Theories that explain consciousness entirely in terms of the content of perception and internal representations are referred to as *first-order representational theories*, whereas *higher-order* theories argue that first-order representations need to be additionally represented (or thought about) in order to be conscious (Dretske, 1995; Rosenthal, 2008; Mehta and Mashour, 2013). Furthermore, Block (1995) proposed that representations that are accessed and used in reasoning and the “rational” control of action should be considered conscious, whether they have phenomenal aspects or not.

Unfortunately, it is difficult to tell what exactly makes perception and internal representation, or the processes in which they are involved, conscious. Indeed, the complex and adaptive perceptual and behavioral capacities of organisms, as well as artificial agents or robots, are normally described and explained without ever using the word *consciousness* (Brooks, 1997; Clancey, 1997; Ziemke and Scharkey, 2001; Sloman and Chrisley, 2005). Furthermore, psychologists increasingly associate human perception and behavior explicitly with the *un*conscious, trying to demonstrate in different ways how “smart” these processes are in the absence of consciousness (Bargh and Morsella, 2008; Dijksterhuis and Aarts, 2010). However, consciousness cannot be sufficiently characterized by studying perceptual and behavioral phenomena that go under the heading of *unconscious*, and by attributing “opposite” qualities to it, such as freedom of choice, free will, or controllability (as opposed to automaticity) or integrative and flexible perception or thinking (as opposed to reacting independently and in parallel to different aspects of the world). As argued later, in light of a different perspective on consciousness, what is usually termed *unconsciousness* may not be the opposite of consciousness, but may refer to something entirely different, namely repeatedly observed, well-learned, and habitual patterns of perception and behavior that can be triggered by impoverished or poorly attended (“subliminal”) stimuli.

Both lay persons and scientists also (and perhaps more strongly) associate consciousness with feelings, sensations, and experiences; subjective or phenomenal aspects that can be primarily described in terms of “what it is like” to have or experience them. The difficulty of addressing these aspects from a third-person or mechanistic perspective reinforces the view that one is confronted with an unbridgeable abyss between mind and brain, an “explanatory gap” or “hard problem” (for general discussions, see Dennett, 1991, 2001; Block, 1995; Chalmers, 2004; Rosenthal, 2008; Velmans, 2009; Blackmore, 2010; Van Gulick, 2011). However, although feelings and sensations are a mystery in an ontological sense, they may refer to a different kind of mystery than consciousness.

In particular, the best way to conceive of feelings caused by physical contact (e.g., hotness, pain, or nausea, or merely feeling that one is touching an object), sensations (e.g., brightness contrast or different tastes, usually not termed *feelings*), or felt impulses or action tendencies associated with motivational states or emotions, is to see them as the relatively direct output of reactive and adaptive sensorimotor or motivational mechanisms that are unconditionally activated by relatively simple features of fitness-relevant objects (see also Zajonc, 1980; Dennett, 1991); with the intensity of the subjective experience non-linearly increasing with the activation level of these mechanisms (Lindsay and Norman, 1977). More useful than to mystify their subjective correlates is to recognize that the underlying mechanisms evolved to force organisms to start doing the right, fitness-promoting thing (e.g., to approach, eat, or flee) under the right circumstances, without yet requiring the organism to have knowledge about the fitness-relevant properties and objects themselves.

From an evolutionary perspective, a “distance sense” such as vision is crucial to anticipate or prepare for physical contact with fitness-relevant objects, allowing the organism to follow and stalk prey and to escape in time from predators. It should be noted, however, that visual perception of objects may be “colored” by feelings and sensations associated with activated motivational mechanisms (James, 1892/1985; Lambie and Marcel, 2002; Frijda, 2005). For example, a particular shape or color of flowers or fruit may look attractive or delicious to a hungry animal, whereas a rapidly approaching strange object causing an escape tendency may be seen as dangerous. Furthermore, although (true) color perception has been the favorite example of philosophers to demonstrate that the experiential aspects of sensations or qualia and hence consciousness have something ineffable (see Dennett, 1991 for a critical discussion), colors may also be conceived as correlates of fitness-relevant properties of objects (e.g., genetic relatedness, edibility, or sexual receptivity) that need to be perceived and discriminated from a distance.

The experience of feelings and sensations is also closely related to attentional processes. For example, when attention is drawn to a particular part of the body due to a sudden increase in sensory stimulation (or attention is directed in a top-down manner to that particular part in order to check to what extent it is still stimulated), the associated sensations or feelings may also increase.

From this view, and a particular perspective on consciousness sketched below, feelings and sensations are neither conscious, nor unconscious. Based on attentional mechanisms that help to potentiate the relevant brain areas, you either have a feeling (and can feel it), or you do not. It is also not meaningful to say that feelings such as pain can “enter” consciousness. As will be argued, when consciousness is interpreted as referring to certain states which themselves are characterized by an absence of feelings and motivated attention, feelings and sensations may be conceived as orthogonal and additive to consciousness.

In addition to their unclear status as aspects of consciousness, feelings and internal representations also tend to be insufficiently distinguished. For example, it is sometimes said that feelings of bodily states (Damasio, 1999) or sensations of contrast or movement (Block, 2009, 2010) “represent” something. However, this refers to an entirely different way of representing than internally representing or remembering objects and their invariant properties. While the former “representation” lasts as long as the relevant senses and projection areas in the brain are stimulated, the latter require sensorimotor networks that somehow (probably in terms of synaptic weights) can store and preserve structural aspects of object properties or dispositions (Sommerhoff, 1974; Grossberg, 1980).

On the other hand, in addition to having affective consequences when activated, representations themselves are sometimes unclearly endowed with experiential or phenomenal aspects (e.g., Searle, 2000), with some arguing for a “cognitive phenomenology” (for a discussion, see Bayne and Montague, 2011).

What is left of consciousness after feelings and sensations have been separated from it, and after we have stopped attributing qualities to it that are assumed to be “opposite” to well-learned and habitual (“unconscious”) patterns of perception and behavior? The answer proposed here is: a capacity for objectivity, to be defined as the capacity to produce *states* of objectivity that internally represent objects and their dispositional properties (as well as movements and behaviors predicted by these dispositions) in relatively stable, accurate, increasingly complete, perceiver-independent and neutral ways, unbiased by specific needs, motives, and anticipation of instrumental aspects and rewards. These states or moments, however, are normally preceded and followed by (and tend to be combined or fused with) sensations and feelings with phenomenal or experiential aspects which are caused by activation of sensory and motivational mechanisms. It may be argued that states of objectivity themselves are associated with a very special phenomenal aspect; the experience that subjective aspects are *absent* and one is “just looking” at the world as it really is and can be. (A more daring interpretation would be that states of objectivity have no experiential aspects at all.)

Associating consciousness with objectivity implies that feelings are different from it and that it is not the opposite of the fast and automatic processes commonly referred to as *unconscious* (these processes may need to be accompanied by moments of objectivity in order to be adaptively matched to the particular situation). Furthermore, this association is quite consistent with views that equate perception and consciousness, but inconsistent with the notion of “unconscious perception.” It is quite likely that much of our behavior is influenced by impoverished and subliminal stimuli. But these stimuli are not perceived as objects in the above sense. Exposure to these stimuli may or may not result in mediating states of objectivity.

A thorough examination of the relationship between consciousness and a capacity for objectivity may be promising for three different reasons. First, the concept of objectivity captures an important everyday intuition about the nature of consciousness that is neither sufficiently described in terms of feelings or sensations, nor perception or internal representation as commonly interpreted. In particular, it is widely recognized that there is something remarkably non-subjective about consciousness, not necessarily implying a particular egocentric perspective or experience of feelings. That is, common sense associates consciousness with accurately recognizing (or “realizing”) the presence or existence of perceiver-independent objects, and a relatively disengaged, integrated, and objective view in which multiple properties of objects can be appreciated from many different perspectives; making us confident that at any time we want to, we can walk toward and around the objects themselves, and touch and manipulate them to check if they really have the perceived or expected properties. Merker (1997) describes this aspect of consciousness usefully in terms of “being in the presence of” and points out that it involves a *relationship* between subject and object (see also Campbell, 2009, 2011). Importantly, the experience that we can “just look” at objects without in any way feeling affected by them, is not sufficiently captured by the concepts of perception and internal representation as currently conceived. Indeed, it seems to require a conceptualization in terms of a general disposition or readiness to look at objects from multiple egocentric perspectives; perspectives that somehow can be momentarily combined into an allocentric or objective representation of the world (e.g., Husserl, 1907/1991; Grush, 2000; Schellenberg, 2007; Campbell, 2009). An important question, of course, is how we should conceptualize that disposition in mechanistic and neural terms.

A second reason for examining more closely the relationship between consciousness and a capacity for objectivity is that empirical research on consciousness already assumes and partially confirms a close link between consciousness and that capacity. This is first evident from researchers operationally defining consciousness as the ability to *accurately* report the presence and nature of physical stimuli and objects (Baars, 1988; van Boxtel et al., 2010). Furthermore, the processes that neuroscientists associate with consciousness often can be interpreted as providing the minimal requirements for accurate or objective perception, such as the recurrent processing between different parts of the brain necessary for relatively stable states that can integrate or “bind” multiple sensory aspects of the same object (e.g., Dehaene et al., 1998; Damasio, 1999; Crick and Koch, 2003; Lamme, 2010). As will be discussed in greater detail in the next section, especially research on the accurate perception of, and iconic memory for, very briefly presented arrays of objects (Lamme, 2010) and natural scenes (Li et al., 2002; Fei-Fei et al., 2007) suggests that brief states of objectivity are possible. Moreover, research on meditation and especially mindfulness suggests that these states can be artificially extended by means of training (Brown et al., 2007; Chiesa et al., 2011).

A third and final reason to consider a capacity for objectivity a central aspect of consciousness is that, from an evolutionary perspective, it would be a tremendously adaptive property of brains. In particular, perceiver-independent representations of the environment and its properties allow organisms to respond in flexible ways to a wide variety of need-relevant situations (e.g., Lorenz, 1954/1984; Bateson, 2005). Indeed, as discussed later, a capacity for objectivity can be alternatively described in terms of the domain-general, decontextualized, and fluid nature of intelligence (Chiappe and MacDonald, 2005). If consciousness is seen as an ultimately adaptive property of brains, then another candidate property such as a capacity for objectivity surely deserves to be compared with it.

It will be argued that a capacity for objectivity not only requires the evolution of cognitive capabilities for integrating different perspectives from which objects may be looked at, but also the evolution of a special motivational mechanism. At first sight, this seems counter-intuitive. That is, objectivity seems to imply the *absence* of motivation and activated needs, and of the associated biased perception of particular instrumental properties or extrinsic rewards. This paper will argue, however, that a capacity for objectivity (and hence consciousness) may be crucially dependent on particular motivational mechanisms that cause individuals to be intrinsically motivated to explore and play with objects, and to behaviorally manipulate them in a *careful* and *thoughtful* manner, treating them as vulnerable. It will be proposed that the motivational mechanism controlling the gentle motor aspects of careful object manipulation and exploration has its evolutionary and social roots in kin selection (Hamilton, 1964). Interestingly, such an evolutionary analysis suggest that a capacity for objectivity may not only be the basis for intelligence and skillful and “fine” manipulation of objects (as required, for example, in tool making), but also for conscience or morality (it is considered good to treat vulnerable objects in an attentive and thoughtful manner), and esthetic experience (vulnerable but “fit” objects are seen as beautiful). The present paper is the first systematic attempt to explain how consciousness in humans, conceived as an especially well-developed capacity for objectivity, might be responsible for the presumably unique human ability to integrate intelligence, morality, and esthetics.

The rest of this paper is organized as follows. The next section examines a number of questions and phenomena that need to be addressed in order to start a fruitful search for the mechanisms underlying a capacity for objectivity. A subsequent section attempts to describe in greater detail the evolution and development of a capacity for objectivity. How a capacity for objectivity is related to intelligence, conscience, and esthetic experience, and contributes to human uniqueness is described in two subsequent sections.

## SEARCHING FOR THE PROXIMATE MECHANISMS UNDERLYING STATES OF OBJECTIVITY

### OBJECTIVITY AS INTEGRATION OF MULTIPLE LOOKS

Different philosophers have used spatial perception and memory as a paradigm for examining how objectivity is possible (e.g., Husserl, 1907/1991; Grush, 2000; Schellenberg, 2007; Campbell, 2009). Although there are differences in approach, a common thread running through these analyses is the assumption that perceivers somehow are able to obtain information about their environment by adopting multiple egocentric perspectives or viewpoints in order to arrive at an allocentric, map-like, or objective representation that can guide their behavior. Objectivity also seems to require that perceivers can imagine to move to another location from which they can look at their current location and understand its unique perceptual consequences (Grush, 2000). The view that spatial memory is based on a combination of viewer-centered updating and viewer-independent representation of geometric properties is well-supported by studies demonstrating the ability of vision-dependent animals to find previously stored food or their way home after disorientation or starting from new locations (Burgess, 2006; Meilinger and Vosgerau, 2010).

The importance of movement in updating was nicely demonstrated in an experiment by Simons and Wang (1998). Participants sat in front of a round table on which an array of five different objects was visible for 3 s. After waiting for 7 s with objects hidden from view and moving around the table to a new viewpoint, they were better able to tell which of the five objects changed location than after an equivalent rotation of the table. The authors explain this improvement in recognition memory in terms of a viewer-centered updating of the original representation; participants build up an expectancy about how their movement will change the appearance of the layout. Importantly, imagining oneself to move to a novel viewpoint has similar beneficial effects (Presson, 1982; Christou and Bülthoff, 1999), suggesting that the information used in updating may not only come from proprioceptive feedback but also from efference signals that would have been required to take a new perspective and that probably activate the relevant sensory brain areas without resulting in muscle innervation (Hesslow, 2010).

Like the perception of space, the perception of individual object properties such as shape, softness, or heaviness can also be conceived as the formation and application of an expectancy that certain changes in sensory input will follow from one’s own movements, change of viewpoint, or manipulations of objects. The correlations between motor output and sensory input may be stored (perhaps in terms of synaptic weights) in sensorimotor networks. For example, in moving one’s eyes to follow the contours of an unfamiliar object, or in changing one’s point of view from which the object is looked at, one produces certain changes in retinal images that are subsequently expected to occur when the same motor output with respect to the object is again generated. Similarly, perceiving an object as heavy means that one expects it to offer relatively much resistance (due to gravitational force) when actually lifted (Dijker, 2008). That perception of properties can be conceived as accurate prediction has been nicely described by von Helmholtz (1878/1971): “Each movement we make by which we alter the appearance of objects should be thought of as an experiment designed to test whether we have understood correctly the invariant relations of the phenomena before us, that is, their existence in definite spatial relations” (p. 384).

Many current theories of perception recognize the importance of sensorimotor processes for extracting and using information about need-relevant properties or affordances of objects (e.g., Sommerhoff, 1974; Ellis and Tucker, 2000; O’Regan and Noë, 2001). These processes may be rooted in a more elementary capacity of the brain to reliably distinguish between changes in sensory input that are caused by self-movement and those that are caused by the environment (Von Holst and Mittelstaedt, 1950; Burge, 2010).

The development or ontogenesis of individual organisms in large part can be described as the acquisition or learning of multiple expectancies about the world and its instrumental properties by means of exploration and behavioral manipulation (Piaget, 1936/1952); with the acquired expectancies increasingly allowing individuals to “go beyond the information given” and to predict and prepare for events, culminating in visual imagery and total “offline” simulation of perception and behavior (Hesslow, 2010). However, it is difficult to understand how acquired expectancies are used in a top-down manner to establish states of objectivity as previously defined. Indeed, there seems to be a contradiction involved here. On the one hand, objectivity tends to be associated with the absence of expectancies and prejudgment; on the other, perception without expectancies seems impossible. This contradiction may be resolved by imagining perceivers to activate simultaneously all possible expectancies associated with all possible viewpoints, and to somehow integrate these into a single neural state which can be described as a readiness (Ryle, 1949/1963) or skill (Grush, 2000; Schellenberg, 2007) for actually adopting the different perspectives if required. Additionally, one could assume that even a single look at an object involves a very brief process of testing (von Helmholtz, 1910/1925) several expectancies about the properties of an object (e.g., confirming its expected shape, size, or distance by means of small movements or merely sending efference signals to sensory projection areas; cf. Hesslow, 2010). Visual imagery and offline simulation of perception and behavior may also continuously support actual perception and behavior, by providing information about the consequences of changes in viewpoints and behavioral manipulations. It becomes increasingly clear that visual imagery not only accurately models the physical properties of objects and spatial layouts (Kosslyn, 1995), but also uses the same sensorimotor structures as perception and behavior in response to real objects (Kosslyn, 2005; Zacks and Michelon, 2005; Decety and Grezes, 2006; Hesslow, 2010). Thus it seems plausible to assume that a “single” look at an object or spatial layout in a rudimentary way involves imagining oneself moving to different locations from which one may look at the object or layout from different perspectives and orientations; or imagining the consequences of manipulating the object in different ways.

### HOW COMPLETE IS A SINGLE LOOK?

Recent research increasingly suggests how accurate, complete, and in an important sense objective, representations of very briefly presented pictures of three-dimensional objects and complex sceneries can be. For example, Lamme and colleagues (for a review, see Lamme, 2010) exposed participants during 0.5 s to a group of line drawings of eight everyday objects. Following a retention period of 1.5 s, they were presented again with the picture and had to indicate which object had been replaced by another. When, during the retention period, the change was preceded by a cue pointing to the location of the to-be-replaced object, participants were quite able to detect the replacement. The researchers attribute this performance to iconic or very short visual memory, which they describe as fragile and easily overwritten, but as having a large capacity (in contrast to working memory). According to Lamme (2010), iconic memory involves perception of objects rather than simple feature detection or a retinal after image. Interestingly, it has been shown that visual illusions that seem to require depth perception such as the Ponzo (Ben-Shalom and Ganel, 2012) and Kanizsa illusion (Vandenbroucke et al., 2012) can also be fully perceived in iconic memory.

Using a wide variety of natural scenes, Fei-Fei et al. (2007) demonstrated that after brief presentations, participants usually are quite able to verbally report the content of these scenes in terms of gist. Using a dual-task paradigm, requiring participants to perform a central attention-demanding task while pictures of sceneries were flashed peripherally for 30 ms, Li et al. (2002) found that the gist of a scenery can be detected under much poorer viewing conditions. Under these conditions, participants can also tell, for example, whether the scenery contains an animal or vehicle. Importantly, when the peripheral task required participants to discriminate between arbitrarily rotated letters, performance appeared to be quite poor, confirming the importance of gist and object perception in quickly forming accurate internal representations.

It is important to note that the richness and accuracy of internal representations of relatively static displays not necessarily implies that perceivers can accurately detect *changes* in these displays (Simons and Rensink, 2005). In particular, research on change blindness amply demonstrates that perceivers who believe to have a full and complete view of a photograph of a scenery, have great difficulty detecting changes in the scenery from one presentation to the next when changes are accompanied by a flicker, preventing attention to be drawn to the change (e.g., Rensink et al., 1997). This is especially true for changed features that are relatively less attention-drawing (Rensink et al., 1997), or less relevant for the gist of a scenery (Sampanes et al., 2008). However, using more subtle and implicit measures of change detection, other researchers (e.g., Fernandez-Duque and Thornton, 2003) have shown that even unnoticed changes may influence the visual system, suggesting that visual representations are richer than studies of change blindness have led us to believe.

Answers to the question to what extent iconic memory is based on attentional processes and hence may involve selective or biased perception, depends on the way attention is conceptualized. For present purposes, it is especially relevant to allow for the possibility that iconic memory may be based on diffuse rather than focused attention, or alternatively, on the very fast switching of focused attention (VanRullen et al., 2007; Marchetti, 2012). In both cases, the end result would be a relatively global and non-selective look at the environment. To the extent that consciousness is associated with states of objectivity produced by diffuse attention, consciousness and focal attention can be seen as independent. That is, after a more global representation of a complex situation involving multiple objects has been established, focal attention may be selectively directed at certain objects for further analysis. This is well described by van Boxtel et al.’s (2010) distinction between the synthetic aspects of consciousness and the analytical aspects of attention. It goes without saying that the global and accurate nature of iconic memory is easily reduced when the different objects perceived are associated with need-relevant and attention-drawing features. Furthermore, as social-psychological research on stereotyping and prejudice has extensively shown, accurate perception is less likely in case of strong needs, strong and emotional stereotypes, ambiguous and complex information, and time pressure (for a recent review, see Fiske and Taylor, 2013).

### SUSTAINING STATES OF OBJECTIVITY THROUGH MINDFULNESS

Recent research suggests that brief moments of objectivity in normal perception may be artificially maintained and extended by means of *mindfulness training*. It is said that “a mindful mode of processing involves a receptive state of mind, wherein attention is kept to a bare registering of the facts observed …[permitting] the individual to ‘be present’ to reality as it is … without overlay of discriminative, categorical, and habitual thought (…)” (Brown et al., 2007, p. 212). Mindfulness has also been described as a balance between a relaxed state and vigilance (Zeidan et al., 2010).

Research on the cognitive consequences of mindfulness training indicates that mindful individuals (1) show less change blindness, (2) identify more alternative figures or perspectives in ambiguous figures, and (3) show less interference from invalid cues in a visual selective attention task (Hodgins and Adair, 2010). Furthermore, they show a smaller attentional blink (i.e., less deterioration of detection of stimuli that are presented in close temporal proximity; Slagter et al., 2007), and a greater ability to stabilize an ambiguous percept in a bistable image paradigm such as the Necker Cube, when instructed to hold a particular perspective (Sauer et al., 2012). In addition, beneficial effects of mindfulness on working memory have been demonstrated (Zeidan et al., 2010).

Most theorists of mindfulness assume an inherent link between objectivity and prosocial tendencies such as empathy, sympathy, and kindness (Brown et al., 2007; Holas and Jankowski, 2013), without, however, explaining where these tendencies come from and how they can be consistent with objectivity. Indeed, it seems a mystery how objectivity can have implications for kindness, except perhaps that it implies the absence of fear, aggression, or any other egocentric motive. This paper argues, however, that the association between mindfulness and prosocial tendencies may point to a crucial role for a particular motivational mechanism in states of objectivity.

To summarize, a capacity for objectivity may have its evolutionary roots in mechanisms that allow organisms to distinguish between sensory changes produced by own movements and by the environment, and that also would underlie elementary perceptual constancies. More complex behavior and object manipulation, however, would require the formation of sensorimotor expectancies about almost any conceivable property or disposition of objects and spatial layouts. These expectancies are the building blocks for complex neural states that at this moment can only be functionally described in subjective terms (e.g., “my current perception is based on my current point of view or a particular manipulation of the object, but I am confident that the same object can be looked at from many different points of view and that I can predict the changes in sensory input that will result from actually adopting these viewpoints”).

This characterization of states of objectivity would be consistent with the current emphasis in consciousness research on the importance of relatively stable neural states of widely distributed neural networks, acting as a kind of workspace for further analysis of perceived objects (Dehaene et al., 1998; Doesburg et al., 2008). However, the present description specifically assumes that changes in these states reflect a covert form of perception and behavior; with the perceiver simulating the perceptual consequences of adopting different points of view or performing different manipulations.

The present view on the importance of states of objectivity is also similar to the one proposed by Merker (2013), who describes a conscious state as “naïve realism” and argues that it is made possible by an integration of information provided by the senses, own movements, and motivational systems. This state allows the organism to be primarily concerned with the objective aspects of its environment and not to be bothered by the sensations that might be produced by underlying perceptual and behavioral mechanisms. The present paper, however, adds to this that a conscious state requires awareness of the possibility of multiple looks or behavioral manipulations, and the inhibition of motivational systems that could bias perception. As argued in the next section, states of objectivity are not only realized by brain mechanisms of a subject trying to make sense of a pre-existing objective world, but also by behavioral attempts to make objects themselves permanent by preserving, protecting, perhaps even constructing and beautifying them. These attempts most likely are motivated and controlled by a specific motivational mechanism with a social origin. To see this, we need to combine a developmental and evolutionary perspective (Tinbergen, 1963).

## A DEVELOPMENTAL AND EVOLUTIONARY VIEW ON A CAPACITY FOR OBJECTIVITY

As described earlier, properties can be learned and internally represented through interacting with objects in a goal-directed and instrumental manner, normally motivated by specific needs or activated motivational systems. It seems possible that, in this way, organisms can acquire extensive internal representations of the multiple ways in which objects can be used, and that the presence of these representations are a sufficient condition for the development of a relatively neutral and disengaged look at objects. However, the formation of these experienced-based and elaborate internal representations takes time, may be dangerous and hence non-adaptive, and does not guarantee that relevant properties are internally represented and available when it is urgent to satisfy needs. Indeed, theorists have suggested that there are ways of acquiring extensive knowledge about objects and their properties that are more adaptive in the long run and that do not require instrumental interaction with objects.

The concepts at stake here are exploration and play. The young animal explores new objects in its environment by manipulating them, subsequently observing “interesting” effects such as sounds or visible reactions. By repeatedly producing these effects, behavior becomes increasingly effective and skillful (Piaget, 1936/1952). It is difficult to deny that young animals are intrinsically motivated to perform these behaviors, as there are no obvious extrinsic rewards (e.g., food, safety) to be obtained *and* they visibly enjoy themselves. This is exactly the reason why theorists associate curiosity and interest with the concept of intrinsic motivation, and an increase in knowledge (Deci and Ryan, 2000) and reduction of prediction errors (Oudeyer and Kaplan, 2007; Gottlieb et al., 2013) with intrinsic rewards.

While the concept of intrinsic motivation suggests that accurate and elaborate internal representations of objects and their properties may be formed through satisfaction of curiosity, it tells us little about the particular quality that exploratory or playful behavior should have in order to contribute to the formation of these representations. In particular, one would expect that this behavior must allow for the extensive testing of hypothesized properties of objects, while at the same time respecting the integrity of these objects through a particular quality of physical contact and manipulation best termed *carefulness* or *thoughtfulness*; as if the vulnerability of these objects is taken into account.

To identify the underlying motivational mechanisms, it is important to distinguish between the vulnerability of the perceiver and the vulnerability of the object, and hence between two different senses of carefulness. In the former sense, objects are “carefully” explored for certain interesting and need-relevant features while keeping in mind the object’s potential dangerousness and one’s own vulnerability. This suggests that a fight-or-flight system (Panksepp, 1998), competing with curiosity, plays a role here. Consistent with this possibility, Lorenz (1981) has described exploration in terms of an approach-avoidance conflict, caused by a competition between a flight response and another self-preservational mechanism such as hunger, causing a relatively fixed distance from which an ambiguous object can be safely observed, cautiously approached, and probed in order to discover its fitness-relevant properties. It has been additionally suggested that the mutual inhibition of attack and flight tendencies is responsible for the restrained, gentle, and ritualized manner in which, for example, cats play with life prey (Pellis et al., 1988)

In addition to treating oneself as vulnerable, treating the object that is explored or played with as vulnerable becomes especially important when these objects are vulnerable kin (e.g., siblings or young offspring), and harming them implies a decrease in inclusive fitness (see below). In that case, the behavior should not only be careful in the sense of fearful but also in the sense of protective, gentle, and conscientious. As discussed below, touching and handling living as well as non-living objects in a gentle and careful manner would be ideally suited for discovering and internally representing the properties of these objects in relatively complete and objective ways. But is it plausible to assume that a mechanism for this behavior has evolved?

It may be proposed that this kind of behavior is motivated by a *care mechanism* (Dijker, 2011, 2014) and that its evolution can be predicted by the genetic cost-benefit model of kin selection or inclusive fitness (Hamilton, 1964). According to that model, not only behavioral mechanisms in the service of self-preservation and reproduction will evolve, but also mechanisms for prosocial behavior. Although this model has been primarily used to study how kinship cues (e.g., color, shape, smell, familiarity) and kin-recognition mechanisms positively affect prosocial behavior (e.g., West et al., 2007; Park et al., 2008), it also allows the prediction that a reactive psychological mechanism will evolve that specifically responds to the property vulnerability and its associated cues, and prevents that harm is done to vulnerable kin (Dijker, 2011, 2014).

From an evolutionary perspective, vulnerability can be defined as the disposition or likelihood of living things to change into a state of lowered fitness (a state inconsistent with their “design specification”) when exposed to certain conditions. For those concerned with the fitness and well-being of others, an assessment of vulnerability would be crucial as information about this property can help them to predict and thus prevent actual harm. After all, especially in ancestral environments, it would have been much better to prevent injury than to try to relieve harm already inflicted and much more likely to result in death. (Of course, predators have other reasons to be interested in the vulnerability of potential prey.)

Cues correlated with vulnerability include relatively small size or weight, transparency or other correlates of fragility, visual, olfactory, or vocal signs associated with young age and immaturity. Signs of actual harm, suffering, or distress may also inform about vulnerability (Dijker, 2014).

The care mechanism that is automatically triggered by these cues normally generates behavior that can immediately improve the vulnerable object’s condition such as impulsive helping or aggressive defense against third parties, yet may also be visible in aggression inhibition in response to the vulnerable object and in treating it gently, carefully, and with foresight.

When all members of a social group, young and old, males and females, are endowed with such a care mechanism, not only the conditions for cooperative group living would be established (Dijker, 2011), but also for acquiring social and technical skills. In particular, social and rough-and-tumble play allows siblings to learn social properties and is made possible by mutually activating a care mechanism by repeatedly showing signs of vulnerability, commonly termed *play markers* such as laughter or smiling, and active demonstrations of self-handicapping (Fry, 2005). The same care mechanism enables adult group members to protect and tolerate the presence of playful and potentially annoying youngsters. This tolerance would also allow the latter to approach adults and observe and imitate their technical skills (Van Schaik et al., 1999).

There is something special about the asymmetrical and responsible nature of parental care of vulnerable offspring. That is, parental care is associated with different subsystems involved in, for example, defense, feeding, cleaning, and teaching (George and Solomon, 1999). Yet it also strongly leans on playfulness. Inclusive fitness would be enhanced if playfulness in young age can be combined with elements of mature and responsible help and care (Hrdy, 2009).

Now consider how a care mechanism, when generally applied to any living and non-living thing that is perceived as vulnerable, helps to acquire elaborate and objective representations of objects and their properties; representations with cognitive, moral, and esthetic aspects. Unlike representations formed on the basis of other motivational systems (including those involved in curiosity and exploration) that do not aim at protecting or improving the fitness of other individuals, the stability and accuracy of care-based representations derive from attempts to make the perceived and internally represented objects *themselves* more permanent, taking into account their vulnerability as in social play and parental care. These attempts make these objects continuously available for observation, exploration, and experimentation, in the course of which new properties and relationships may come to the fore. From the perspective of kin selection, the state of relative permanence or fitness is desirable, and the different behaviors performed to care for vulnerable siblings or offspring are the right things to do and morally good. Furthermore, caregiving behavior may involve cleaning, reparation, restoration, and different kinds of maintenance activities, aimed at improving an organism’s health and fitness (in agreement with “design specifications”), the result of which may be perceived as beautiful. These esthetic aspects of the acquired representation, in turn, make care objects more attractive and attention-grabbing, thereby increasing the motivation to manipulate them in gentle and protective ways, resulting in representations that are still richer in detail and more objective. Importantly, in the course of this process, keeping the object in mind and looking at it from multiple perspectives (if neurally possible) is as important as ensuring that the object actually remains present and in good condition. (At a new level, an organism may be able to objectify its own body, represent it as vulnerable, and also treat it in careful and protective ways.)

According to this account, knowledge acquisition not only is associated with intrinsic motivation (the common view of curiosity and play), but also with a special kind of extrinsic motivation when objects are perceived as vulnerable. The extrinsic rewards are obtained by observing an increase or growth in the manipulated object’s physical or mental condition (evidence for fitness) after treating it in a protective and fitness-promoting manner. The intrinsic rewards are associated with the satisfaction or pleasure being felt on the basis of these observations. As a result, care objects are manipulated in such a way that many of their properties can be discovered and internally represented from multiple perspectives.

## OBJECTIVITY, WITH FEELING AFTER ALL?

It is a counter-intuitive and interesting result of the present analysis that a state of objectivity would be associated with esthetic experience. Does not this make this state less objective? Yet, it is the nature of the object-focused feeling involved that may make the association less strange. According to the present view, activation of a care mechanism in response to a vulnerable yet healthy or “fit” object, showing agreement with its “design specifications,” causes a distinct motivational state or emotion: tenderness. Tenderness is an emotion that has received much attention from eighteenth and nineteenth century moral philosophers but has disappeared from twentieth century psychology (but see McDougall, 1923/1948). However, it has recently reappeared to explain moral emotions (Dijker, 2010; Lishner et al., 2011) and the experiential effects of oxytocin in parental care (Uvnäs-Moberg, 1998; Dijker, 2014).

There are different reasons why it is difficult to recognize tenderness as a discrete emotion with distinct behavioral manifestations. First, tenderness tends to be exclusively associated with parental (often maternal) care giving and apparently not common in everyday life. Second, in contrast to motivational states more typically discussed in the emotion literature (e.g., fear, anger, happiness, or sadness), tenderness has relatively unremarkable features, not easily recognized in expressive behavior. Third, when recognized as an aspect of prosocial behavior, it tends to be mislabeled as “empathy” (e.g., Batson, 1998; de Waal, 2008) and associated with almost any type of “helping behavior” in response to the suffering or need states of others, making it difficult to associate it with a specific quality of motor output.

Currently the strongest evidence that tenderness is associated with gentle motor output and muscle relaxation is offered by studies showing how this emotion is associated with the hormone oxytocin: a hormone released by a paraventricular part of the hypothalamus, and acting on the parasympathetic nervous system. Its effects include a fall in blood pressure and cortisol levels, and inhibition of flight and fear (Panksepp, 1998), thereby explaining typical experiential aspects of tenderness such as calmness, openness, and relaxation. Importantly, tender responses and their neurophysiological correlates can be observed in both parents and non-parents responding to infantile features, and have been shown to affect a wide variety of non-parental prosocial behaviors (Bartz et al., 2011).

That tenderness and esthetic experience may be closely related, was first described by Burke (1759/1990), who proposed that beautiful things not only tend to be relatively small, smooth, soft, lightly colored, and delicate, but also arouse distinct physiological reactions. He attributed a different kind of esthetic experience to things that are perceived as “sublime” and which are relatively large, hard, dark, irregular, and are fear-arousing, but which can be experienced under safe viewing conditions. Yet, Burke (1759/1990) did not explain how the sublime can be pleasurable. Perhaps, any object that can activate and satisfy another motivation system than care, can be perceived as beautiful as long as it is represented by a state of objectivity and treated as something vulnerable and to be protected. For example, the powerful, threatening, and adapted properties of a dangerous predator may only be judged as beautiful or “fit” (sublime) when objectively represented as something to be preserved. Similarly, while an object that activates the sexual system is appraised as sexy and desirable, it may turn into something beautiful (erotic) when one tries to objectively represent it (Scruton, 2011).

The upshot of all this is that esthetic experience and tenderness, in contrast to other emotions, may not be inconsistent with the notion of objectivity as they imply an exclusive focus on objects themselves, openness to acquire new knowledge about them, and absence of explicit judgment. A similar view is captured by Kant’s description of esthetic experience as *disinterested interest* (Scruton, 2011). Esthetic experience in the full sense may not always accompany states of objectivity, but may always lurk in the background, easily induced in full intensity in particular circumstances or by artists wanting to cause it for different purposes (for a discussion of the different functions of art, see Dissanayake, 2008). As discussed below, another important reason to associate states of objectivity with a reactive motivational mechanism responsible for the arousal of the emotion of tenderness is, that it helps to explain the close connection between objectivity and accurate or “fine” motor skills involved in art, craft, and technology.

Modern cognitive accounts of esthetic experience are not inconsistent with the present view as they associate the perception of beauty with ease of information processing, facilitated by stimulus features such as symmetry, smoothness of lines, repetition, familiarity, or caricature (Lindell and Mueller, 2011). These features, which tend to correlate with marks of physical fitness and health, may indeed help the perceiver to acquire a stable, relatively complete, and objective internal representation of objects.

To summarize, a state of objectivity can be described as a combination of carefulness, tenderness, openness, perception of beauty, and vigilance, together with efforts to keep an object in mind as completely, varied, and detailed as possible, or reconstructing it when noticing that something is missing. Perhaps, a capacity for objectivity and its foundation on a care mechanism are the key to the century-old philosophical puzzle of how judgments of truth, moral goodness, and beauty are related.

## A CAPACITY FOR OBJECTIVITY AS A COMMON BASIS FOR INTELLIGENCE, ESTHETIC EXPERIENCE, AND CONSCIENCE

More than the traditional interpretation of consciousness in terms of phenomenal or representational aspects, an interpretation of consciousness in terms of objectivity allows us to appreciate its functional importance. In particular, it is argued that a capacity for objectivity forms the common basis for achievements in three different but closely related domains: (1) intelligent problem solving, creativity, skillful tool making and use, and technology; (2) art and craft on the basis of esthetic experience; and (3) a combination of different social behaviors enabling relatively peaceful group living, aggression inhibition, and cooperation, and which seem only possible on the basis of a strongly activated care mechanism. In what sense humans are unique in these respects is examined in a subsequent section.

### INTELLIGENCE, CREATIVITY, AND TECHNICAL SKILLS

States of objectivity are necessary for the kinds of problem solving that we tend to consider intelligent and creative. When in a state of objectivity, one tries to be as complete and skilled as possible, by looking at objects from multiple perspectives and performing small, virtual what-if experiments, thereby coming to understand or “grasp” the many relationships among objects and their properties that are possible. When a particular goal or problem comes by, these states ensure that the data necessary for the proposed solutions or decisions are already available (they only need to be “looked at” in new ways), and that solutions will be effective and not based on mere phantasy.

To illustrate, briefly consider an experiment performed with crows to demonstrate how previously acquired knowledge of object or tool properties and corresponding skills are used in a novel context, suggesting relatively perceiver-independent or objective internal representations. Taylor et al. (2010) presented their crows with a box containing meat that could only be obtained by inserting a stick of sufficient length through a hole. However, such a stick was visibly contained in another box and could only be reached by using a shorter stick (a metatool, itself unsuitable to reach for the meat) that was hanging by a string from a branch. Thus participants first had to pull up the string to solve the problem of getting at the food. After receiving training for several of the component tasks (e.g., string pulling for obtaining the short and by itself non-functional stick, using the long stick for extracting the meat), participants appeared quite able to solve the problem. The authors attribute this to a complex cognitive ability involving knowledge of abstract causal rules. However, it is very difficult to imagine how this ability is possible without the birds having acquired a perceiver-independent and objective representation of the total configuration of objects and their individual but interrelated physical properties (see also Köhler, 1917/1957). What seems equally important is a relaxed but vigilant and open-minded state since the animals have good reasons to primarily focus on the food itself and their inability to get at it.

As traditionally conceived, the concept of intelligence does not refer to the content, let alone objectivity, of representations but to the executive (speed, efficiency) aspects and capacity limitations of working memory, measured by common IQ tests (Ackerman and Heggestad, 1997; Conway et al., 2003). However, an interpretation of intelligence in terms of fluidity, decontextualized, and domain-general thinking (Chiappe and MacDonald, 2005) would be quite consistent with the present notion of states of objectivity. Furthermore, although relatively small, correlations between measures of intelligence and the Big Five factors of personality (Costa and McCrae, 1992) also suggest a relationship with states of objectivity. For example, intelligence is positively correlated with the factor Openness to experience (Ackerman and Heggestad, 1997; Chamorra-Premuzic and Furnham, 2006), a factor that is itself associated with esthetic experience (DeYoung et al., 2007). Furthermore, intelligence is negatively correlated with the factor Conscientiousness. Although this correlation has been interpreted as indicating that low intelligence is compensated for with more effortful and elaborate, yet slower thinking (Chamorra-Premuzic and Furnham, 2006), it is equally consistent with conscientious people *wanting* to think objectively, carefully, integratively, and hence more slowly. Furthermore, although a negative correlation between Neuroticism and performance on intelligence tests has been explained in terms of test anxiety, it may also indicate that a chronically activated fight-or-flight system harms the acquisition of objective representations. Finally, the fact that intelligence tests are uncorrelated with the factor Agreeableness may indicate that intelligence can be used for both agreeable (altruistic) and selfish motives, or for solving difficult problems that may be associated with frustration and aggression.

A final observation about the relation between states of objectivity and intelligence is that these states may contribute to the executive aspects of working memory by enlarging attentional span and inhibiting attention to irrelevant information (see the research on mindfulness, discussed earlier).

Whereas intelligence is associated with convergent, problem-based thinking, creativity is associated with divergent thinking. However, it seems wrong to associate creativity primarily with a capacity to make as much combinations as possible between properties that come to mind during “brainstorming.” Indeed, creative thinking seems highly dependent on having thoroughly and repeatedly explored one’s internal representations of the relevant materials (Boden, 1990); following the many “implications” of properties or dispositions of objects already internally represented, understood, and accepted as possible. Original contributions to problem solving are more likely after “taking distance” or “letting it rest,” and looking at one’s internal representation again in a more disengaged, objective, relaxed, and playful manner, undistracted by the different and probably conflicting needs and goals activated during initial exploration. During this phase, one may “suddenly” arrive at a particularly clear view or solution (Lindsay and Norman, 1977). Such a view may actually be consistent with research suggesting that, under particular conditions, unconscious rather than conscious thinking results in high decision quality. For example, Dijksterhuis et al. (2006) found that in case of a complex decision, a group distracted after exposure to the problem and relevant information performed better than a group invited to continue to think about the problem. Although they believe this to be due to unconscious problem solving in the distracted group, Waroquier et al. (2010) attribute it to the high quality of the initial solution, and a deteriorating influence of extensive and conflict-arousing deliberation after the initial solution in the undistracted group. One may additionally propose that distraction enables perceivers to look at the original problem in a new and dispassionate way when they have to come up with their final decision.

Sensorimotor networks involved in states of objectivity not only internally represent properties of objects and help to solve problems; they are also used for effective and skilled interaction with these objects (Ellis and Tucker, 2000), thereby facilitating tool making and technology. This is addressed next.

### TOOL MAKING AND ART ON THE BASIS OF ESTHETIC EXPERIENCE

Theorists tend to emphasize that the mental and behavioral activity of tool makers is controlled by a mental representation of the design or blueprint of the finished artifact, together with explicit and detailed descriptions of the different steps to be followed in order to ensemble it from its different components. Complex tool making, therefore, tends to be associated with a capacity for symbol use (in particular for language based on combinatory rules or syntax; cf. Gibson and Ingold, 1993), enhanced working memory (Wynn and Coolidge, 2007), and sensorimotor skills necessary for effective hand-eye coordination (Wiesendanger, 1999; Byrne, 2004). Although this view recognizes that tool making is guided by the anticipated purpose or instrumental value of a tool, it ignores certain crucial motivational, emotional, and experiential aspects of tool making, especially in relation to humans. In particular, humans differ from the few other tool-making species in that they not only pay more attention to design and complexity, but also show a much weaker tendency to abandon their tools after use. Indeed, they preserve, clean, and repair them and even show a tendency to beautify, decorate, and become emotionally attached to them; the earliest human tools show evidence of beautification and decoration (Balter, 2009).

A state of objectivity integrates esthetic experience, tenderness, care, and specific motor aspects. Hence there may be a close association between making beautiful things (art), craft, and tool making. In particular, during initial stages of tool making, the tool is perceived as a vulnerable object that needs to be treated with care and brought into a less vulnerable and more mature shape by allowing it to “grow” or develop according to its inherent material properties, with the tool maker facilitating this with a gentle and protective attitude (involving activities such as cleaning, polishing, inspecting, touching, testing, and reshaping).

Humans value artifacts that can be recognized as having been crafted and finished with the appropriate nurturance, dedication, and love (Sennett, 2008), and which can be judged in terms of the same moral values and norms that are applied to social behavior. For example, the same words that are used to describe and morally judge the quality of care can also be used to evaluate the manual production and treatment of human artifacts. Thus the cognitive and behavioral activity involved in both nurturing of children and tool making can be described as more or less careful, conscientious, reliable, dedicated, thoughtful, precise, honest, and loving. In contrast, choosing an easier and less risky way of production (e.g., by relying on machines for mass production) or using “cheap” or self-interested motives in making works of art, are negatively evaluated (Sennett, 2008; Bloom, 2010).

### CONSCIENCE, MORALITY, AND SOCIAL SKILLS

It is reasonable to expect that, if a care mechanism would be involved in non-social behavior and intelligence, it must certainly be visible in important social behaviors and phenomena, especially those that are related to peaceful group living, aggression inhibition, and cooperation. First consider the concept of conscience which is much older than that of consciousness. It may be proposed that the concept refers to the accurate or objective perception of a vulnerable object (i.e., to being conscious of a vulnerable object), activation of a care mechanism, and perception or anticipation of the different negative consequences of one’s own behavior for the object’s well-being or fitness, typically experienced as the emotion of guilt. Thus while tenderness is a response to observing that a vulnerable object is in the desirable state of good health, guilt implies the causal attribution to the self of an observed or anticipated decrease in health. Other moral emotions more strongly focus on the harmful behavior of third parties (e.g., moral anger) or the undesirability of the object’s lowered fitness and suffering (e.g., sympathy; see Dijker, 2014 for discussion and comparison with other theories of morality).

Secondly, many social behaviors used for aggression-inhibition, reconciliation, and tolerance seem to rely on activation of a care mechanism which can be activated by mutual displays of vulnerability. This seems to be true for the touching, hugging, playfulness, grooming, gift exchange, shedding of tears, and humor that have been documented in greeting, departure, and politeness rituals (Eibl-Eibesfeldt, 1989). All these behaviors, which have both childish and care-giving elements, are also displayed among friends and lovers (Eibl-Eibesfeldt, 1989).

Two final observations with respect to the role of a care mechanism and states of objectivity in social behavior may be made. First, understanding another person’s viewpoint can be considered a normal aspect of states of objectivity, as these states allow us to imagine ourselves occupying that viewpoint and perhaps even moving toward the person’s location (this would correspond to a cognitive interpretation of empathy). Sometimes, this may result in corresponding feelings. Yet, we may also immediately recognize the other person as a vulnerable being, thereby feeling tenderness (which is not the same as empathy; Dijker, 2010), and perhaps also other moral emotions, dependent on the situation (Dijker, 2014). As group members are endowed with the same capacity for objectivity and share a common environment, their agreements and “intersubjectivity” should not be surprising. Of course, they may still differ widely with respect to needs and interests.

Second, following from the last remark, the present emphasis on a care mechanism does not deny the important role of self-preservational and reproductive motives in interpersonal relationships and society. Indeed, it could be argued that the selfish and manipulative application of intelligence, sometimes referred to as *Machiavellian intelligence* (Whiten and Byrne, 1997), increases in effectiveness when based on a capacity for objectivity developed during a long childhood and partly under the control of a care mechanism. However, the existence of cultures in which power and military skills are praised and vulnerability is devalued, as well as the strong attention of social scientists to competitive and distrustful elements in social interaction (Boehm, 1999; Dijker, 2011), may easily obscure the importance of a care mechanism in human behavior.

## CONVERGENT EVOLUTION OF A CAPACITY FOR OBJECTIVITY AND HUMAN UNIQUENESS

That a capacity for objectivity may depend on a strongly developed and easily activated care mechanism is suggested by the co-occurrence of the two in exceptionally intelligent species that are not genetically related. In these species, the role of a care mechanism would be evidenced by strong parental investment, offspring remaining vulnerable for a relatively long period of time, alloparenting, intense play in both juveniles and adults, and different social behaviors that take vulnerability into account such as aggression-inhibition, mutual helping and protection, tolerance, and reconciliation. For example, both crows and apes in which these behaviors can be frequently observed, also show abundant evidence for curiosity and exploration, spatial memory, tool making and tool use, and intelligent problem solving (Emery and Clayton, 2004).

From similar observations on the behavior of cooperative breeders, some theorists conclude that altruism and social tolerance must play important roles in the evolution of the exceptional intelligence, especially by facilitating social learning, mind reading, and transmission of cultural knowledge (Tomasello et al., 2005; Burkart et al., 2009). However, in addition to this interpersonal facilitation, what is claimed in the present paper is that there is a more intrinsic connection between intelligence and care that allows each group member individually to acquire objective knowledge. As suggested earlier, what is commonly called empathy or intersubjectivity may be derived from this capacity.

Humans are special among the primates in that they can combine a primate capacity for objectivity with a set of unique physical and behavioral traits, allowing for sustained states of objectivity. In motivational terms, humans accomplish this by having an exceptionally strong care mechanism, easily activated by the slightest evidence for vulnerability and immaturity (Lorenz, 1943; Berry and McArthur, 1986; Kringelbach et al., 2008). This sensitivity may be conceived as an adaptation to giving birth to altricial babies (partly due to the smaller birth channel of bipedal humans; cf. Smith and Tompkins, 1995) which remain dependent on others for an exceptionally long period of time, and which requires alloparenting (Hrdy, 2009). Complementarily, due to an evolutionary arms race (Dawkins and Krebs, 1979), human children may have evolved multiple physical and behavioral traits resulting in an increasingly effective trigger for a care mechanism; a “superstimulus” keeping its effectiveness during slow human maturation far into adulthood. This process is complemented with an opposite tendency to acquire certain adult traits (e.g., responsible helping of dependent siblings) quite early in ontogenesis (Hrdy, 2009; Warneken and Tomasello, 2009). In combination, these processes result in the curious human phenomenon that adult caregivers show childish behavior and children signs of mature caregiving (Eibl-Eibesfeldt, 1989; Hrdy, 2009).

In addition to a strong motivational basis for the evolution of a capacity for objectivity, humans also have the physical and neural requirements to form increasingly accurate and complete sensorimotor representations of objects and their properties due to bipedalism, free hands suitable for both a power and precision grip (Marzke, 1996), and an enlarged brain. Combined with a strong and easily activated care mechanism, these traits allow them to more extensively manipulate, explore, and form experienced-based expectancies about objects, as well as to actually make, beautify, repair, and preserve artifacts and tools.

To summarize, a capacity for objectivity may have evolved several times through convergent evolution, but only in combination with uniquely human traits such as exceptional care and free hands, may have resulted in a capacity to form neural representations of objects that have an exceptionally high level of accuracy and objectivity, ready for creative use when the need arises. However, the uniqueness of humans may not derive from a capacity for objectivity *per se*, but from adding language to a capacity for objectivity that in primates had already been developed to an exceptional degree. Language is in two ways related to objectivity. First, language can only be adaptive when it is based on accurate and increasingly complete internal representations of the world; representations that ensure that language has reliable semantic properties. Second, language improves objectivity by creating distance from, and a propositional and impartial view of, the world, allowing us to think *about* objects without attempting to influence or change them. (Of course, on the basis of propositional thought, multiple speech acts, with different instrumental purposes, are possible.)

## CONCLUSION

The main goal of this article was to examine to what extent the concept of objectivity contributes to a better understanding of the nature, functions, and evolution of consciousness. Although the proximate mechanisms responsible for states of objectivity have been barely addressed, a focus on objectivity already appeared useful for clarifying central conceptual problems in the study of consciousness. In general, the concept made it possible to distinguish between objectivity, feelings, and habitual patterns of perception and behavior, and to ask if feelings are an essential element of consciousness, and if “unconscious” habits are the opposite of consciousness. The concept of objectivity has also allowed us to examine how consciousness, to the extent that it refers to states objectivity, is related to other important psychological concepts and their behavioral manifestations. It was concluded that a capacity for objectivity would be an extremely adaptive property of the brain, not only facilitating intelligent problem solving and the acquisition of technical skills, but also group living, cooperation, and morality. Although the adaptive value of elaborate and integrated internal representations, reasoning, mental simulation, or mental time travel is widely acknowledged, almost no systematic attention has been paid to the problem of objectivity.

With respect to the conceptual advantages, the analysis has revealed that states of objectivity should be distinguished from feelings and well-learned or habitual patterns of perception and behavior. It was argued that states of objectivity may be experienced in the highly unique sense of experiencing that one is not affected by the object one perceives, yet that these states may be combined with feelings (e.g., esthetic experience and the perception of beauty). Others might object to the modest role of feelings in explaining consciousness, maintaining that the whole notion of consciousness necessarily implies an experience of being affected by the world around us. However, it is recognized here that states of objectivity normally are closely preceded and followed by, and hence likely to be fused with, feelings. Furthermore, when in a state of objectivity, feelings may be aroused by focusing on particular goal-relevant properties of an object or situation, with objects being selectively appraised as, for example, sexy, delicious, threatening, or kind. When these properties remain standing out and are not appreciated in a more distant manner (perhaps as beautiful), their appraisals tend to reduce objectivity. Indeed, it may be proposed that emotions represent states in which individuals are largely biased, while strongly believing that they perceive the world as it is (in anger, the other person is essentially wrong or bad, in fear, the world is essentially dangerous; cf. Frijda, 2005). While modern emotion theorists strongly focus on the role of evaluative appraisals in emotion experience, they may not have sufficiently realized that it is the unique combination of a capacity for objectivity and evolutionary very old motivational systems (Panksepp, 1998) that is responsible for this strange experience.

The main claim made in the present paper is that brief states of objectivity without feeling not only are possible but also necessary in order to account for intelligent and adaptive behavior. In addition, these states may be considerably prolonged under special circumstances or as a result of training. Saying that consciousness is the same as information integration within a workspace (Baars, 1988; Dehaene et al., 1998) is not enough to characterize the adaptive properties of consciousness, as it tells us little about how integration relates to accurate perception and objectivity.

Now consider research on so-called “unconscious” or automatic influences on perception and behavior, having shown that people are influenced in multiple ways by subliminal, subtle, and impoverished stimuli that can activate habits and reactive sensorimotor mechanisms, subsequently resulting in adaptive, goal-directed behavior, often without requiring states of objectivity (although, as argued, these states may be necessary to regularly check if the mechanisms are activated under the right circumstances). Is there something in consciousness that implies the absence of automaticity or reactivity and the existence of a “free” agent being in control? Saying that in consciousness we have more available information or alternatives to choose from, will not do if one does not tell what makes choosing more free than in the unconscious case. Fortunately, the concept of objectivity may offer an important clue about the meaning of freedom and control. When in a state of objectivity, one has the impression that, given one’s disengaged and open outlook on the world, all courses of action are possible, without feeling urged to choose among them. In contrast, little freedom is experienced when motivational systems are strongly activated, attention is constrained, and a behavioral response is urgent. Intermediate levels of freedom may be experienced when goals are considered relevant but not strongly linked anymore to activated motivational systems of needs.

Many theorists associate consciousness with hypothesis-testing and a functional role in producing adaptive behavior (e.g., von Helmholtz, 1910/1925; Marcel, 1983; Gregory, 2005). James (1892/1985) even proposed an ideo-motor theory according to which a conscious image or thought, after exerting effort to keep it in mind, and in the absence of competing images or thoughts, directly results in behavior; without requiring additional “ will-force” or fiat to execute the behavior. While recognizing these pragmatic aspects, the present paper has argued, however, that in so far as states of objectivity are possible, they would not have direct behavioral implications, and require additional activation of reactive motivational systems in order to cause behavior.

If one is willing to accept the present interpretation of consciousness, it would be a major challenge for future research to specify the mechanisms underlying states of objectivity. As suggested, this research would profit from a focus on mechanisms allowing perceivers to adopt and integrate multiple perspectives, and on developmental aspects of curiosity, play, and care. Although a very demanding research enterprise, it is more likely to be successful than sticking either to a view that associates consciousness exclusively with experiential and subjective aspects, or to one that insufficiently makes clear how perception and internal representation are related to states of objectivity.

## Conflict of Interest Statement

The author declares that the research was conducted in the absence of any commercial or financial relationships that could be construed as a potential conflict of interest.
